# Peritumoral and intratumoral radiomic features predict survival outcomes among patients diagnosed in lung cancer screening

**DOI:** 10.1038/s41598-020-67378-8

**Published:** 2020-06-29

**Authors:** Jaileene Pérez-Morales, Ilke Tunali, Olya Stringfield, Steven A. Eschrich, Yoganand Balagurunathan, Robert J. Gillies, Matthew B. Schabath

**Affiliations:** 1 Department of Cancer Epidemiology, H. Lee Moffitt Cancer Center and Research Institute, Tampa, FL USA; 2 Department of Cancer Physiology, H. Lee Moffitt Cancer Center and Research Institute, Tampa, FL USA; 3 Institute of Biomedical Engineering, Bogazici University, Istanbul, Turkey; 4 Department of Biostatistics and Bioinformatics, H. Lee Moffitt Cancer Center and Research Institute, Tampa, FL USA; 5 Department of Thoracic Oncology, H. Lee Moffitt Cancer Center and Research Institute, 12902 Magnolia Drive MRC-CANCONT, Tampa, FL 33612 USA

**Keywords:** Lung cancer, Biomarkers

## Abstract

The National Lung Screening Trial (NLST) demonstrated that screening with low-dose computed tomography (LDCT) is associated with a 20% reduction in lung cancer mortality. One potential limitation of LDCT screening is overdiagnosis of slow growing and indolent cancers. In this study, peritumoral and intratumoral radiomics was used to identify a vulnerable subset of lung patients associated with poor survival outcomes. Incident lung cancer patients from the NLST were split into training and test cohorts and an external cohort of non-screen detected adenocarcinomas was used for further validation. After removing redundant and non-reproducible radiomics features, backward elimination analyses identified a single model which was subjected to Classification and Regression Tree to stratify patients into three risk-groups based on two radiomics features (NGTDM Busyness and Statistical Root Mean Square [RMS]). The final model was validated in the test cohort and the cohort of non-screen detected adenocarcinomas. Using a radio-genomics dataset, Statistical RMS was significantly associated with *FOXF2* gene by both correlation and two-group analyses. Our rigorous approach generated a novel radiomics model that identified a vulnerable high-risk group of early stage patients associated with poor outcomes. These patients may require aggressive follow-up and/or adjuvant therapy to mitigate their poor outcomes.

## Introduction

The National Lung Screening Trial (NLST) demonstrated that annual screening with low-dose helical computed tomography (LDCT) compared to chest radiography is associated with a 20% relative reduction in lung cancer mortality among high-risk individuals^1^. However, LDCT screening can lead to overdiagnosis and overtreatment of slow growing, indolent cancers that may pose no threat if left untreated^2,3^. Prior *post-hoc* analyses of the NLST have estimated that 18–22.5% of screen-detected cancers would not become symptomatic in a patient’s lifetime and would remain as indolent lung cancer^4^. At present there is limited data regarding the potential harmful impact of overdiagnosis on lung cancer outcomes; however, studies have suggested overdiagnosis is associated with increased operative mortality, severe disability among survivors, and reduction in longer term disease-free survival attributed to loss of pulmonary reserve^5^. Though clinical guidelines provide recommendations for the management of screen-detected nodules, there are limited tools to discriminate between indolent and aggressive lung cancers diagnosed in the lung cancer screening setting^6–9^. As such, biomarkers that can classify behavior of screen-detected lung cancers is an unmet clinical need since prior studies have suggested that 10 to 27% of lung cancers are over-diagnosed in lung cancer screening^10–13^.

Quantitative image features, also known as radiomics^14^, are non-invasive biomarkers that are generated from medical imaging and reflect the underlying tumor pathophysiology and heterogeneity. Radiomics have many advantages over circulating and tissue-based biomarkers as they are rapidly calculated from standard-of-care imaging and they reflect the entire tumor burden and not just a sample of the tumor in the case of tissue-based biomarkers. Our group^8,15–17^ and others^18–20^ have utilized radiomics in the lung cancer screening setting to improve risk prediction and diagnostic discrimination. To date, there have been limited efforts to use radiomics to predict tumor behavior and patient outcomes in the lung cancer screening setting.

Using publicly available data and LDCT images from the NLST, we generated radiomic features from screen-detected, incidentally-diagnosed lung cancers. Radiomic features describing size, shape, volume, and textural characteristics were calculated from the intratumoral region (area within the tumor) and from the peritumoral region (area surrounding the tumor parenchyma). The goal of this study was to utilize these peritumoral and intratumoral radiomics to identify a reproducible parsimonious model that predicts survival outcomes among lung cancer patients diagnosed in the lung cancer screening setting.

## Results

### Patient characteristics

There were no statistically significant differences between training and test cohorts for age, sex, smoking status, number of pack-years smoked, family history (FH) of lung cancer, histology, treatment, stage, and baseline screening result (Table 1). Self-reported chronic obstructive pulmonary disease (COPD) was significantly higher among patients in the test cohort versus the training cohort (16% vs. 7%, *P* = 0.02). Using Student *t* test, we found no statistically significant difference in mean age between training and test cohort when stratifying then among male (*P* = 0.99) and among females (*P* = 0.73).

**Table 1:** Patient characteristics in the training and test cohorts.

Characteristics	Total (N = 234)	Training Cohort (N = 161)	Test Cohort (N = 73)	*P* value^1,2,3^	Validation Cohort (N = 62)
Age, mean (sd)	63.8 (5.1)	63.9(5.1)	63.5 (5.3)	0.63	67.2 (9.47)
By sex					
Female	36.0 (5.2)	63.2 (5.2)	62.8 (5.2)		67.73 (8.92)
Male	64.3 (5.0)	64.3 (4.9)	64.3 (5.3)		66.58 (10.10)
Sex, N(%)				0.06	
Female	101 (43%)	63 (39%)	38 (52%)		30 (48.4%)
Male	133 (57%)	98 (61%)	35 (48%)		31 (50%)
Missing					1 (1.6%)
Smoking status, N(%)				0.82	
Former	100 (43%)	68 (42%)	32 (44%)		59 (95.2%)
Current	134 (57%)	93 (58%)	41 (56%)		0(0%)
Never					2 (3.2%)
Missing					1 (1.6%)
No. pack-years, mean (SD)	64.7 (23.9)	64.5 (23.6)	65.1 (24.9)	0.86	n/a
FH of lung cancer				0.74	
No	170 (73%)	118 (73%)	52 (71%)		n/a
Yes	64 (27%)	43 (27%)	21 (29%)		n/a
Self-reported history of COPD				0.02	
No	211 (90%)	150 (93%)	61 (84%)		n/a
Yes	23 (10%)	11 (7%)	12 (16%)		n/a
Histology				0.23	
Adenocarcinoma-BAC^4^	130 (56%)	96 (60%)	34 (47%)		55 (88.7%)
Squamous	44 (19%)	28 (17%)	16 (22%)		0 (0)
Small Cell	10 (4%)	5 (3%)	5 (7%)		0 (0)
Other NOS	50 (21%)	32 (20%)	18 (25%)		0 (0)
Missing					7 (11.3%)
Treatment				0.22	
Surgical resection	182 (78%)	129 (80%)	53 (73%)		n/a
Chemotherapy/Other	21 (9%)	11 (7%)	10 (14%)		n/a
Radiation therapy	31 (13%)	21 (13%)	10 (14%)		n/a
Staging				0.70	
I and II	179 (76%)	122 (76%)	57 (78%)		45 (72.6%)
III and IV	55 (24%)	39 (24%)	16 (22%)		17 (27.4%)
Baseline Screening				0.19	
Positive (T0+)	158 (68%)	113 (70%)	45 (62%)		n/a
Negative (T0−)	76 (32%)	48 (30%)	28 (38%)		n/a
5-year overall survival rate **(%)**	64%	62.60%	56.20%	0.44	64%

Abbreviations: SD = standard deviation; FH = family history; Pack-years = packs smoked/day x years smoked; COPD = chronic obstructive pulmonary disease; NOS = not otherwise specified;

^1^ P value obtained from Chi-squared for categorical variables.

^2^ *P* value obtained from T-test for continuous variables.

^3^ *P* value obtained from Log-rank for survival variables.

^4^ BAC and adenocarcinoma were combined into one group.

### Radiomic analyses

A total of 109 peritumoral features were extracted, of which 56 were found to be both stable and reproducible, and a total of 155 intratumoral features were extracted, of which 35 were stable and reproducible. Therefore, a total of 91 stable and reproducible radiomics features (peritumoral and intratumoral) were subjected to univariable analysis. In univariable analyses, 40 of the 91 radiomic features (26 peritumoral and 14 intratumoral) were significantly associated with OS in the training cohort (Supplemental Table 1) and 30 of the 40 features were eliminated because they were correlated. The 10 remaining features were reduced to four highly informative features using backward elimination (Supplemental Table 2). Among the four features, three were peritumoral (average co-occurrence joint entropy, NGTDM busyness, and average co-occurrence angular second moment) and one was intratumoral (statistical root mean square).

### Classification and regression tree (CART) analysis

**Figure 1:**
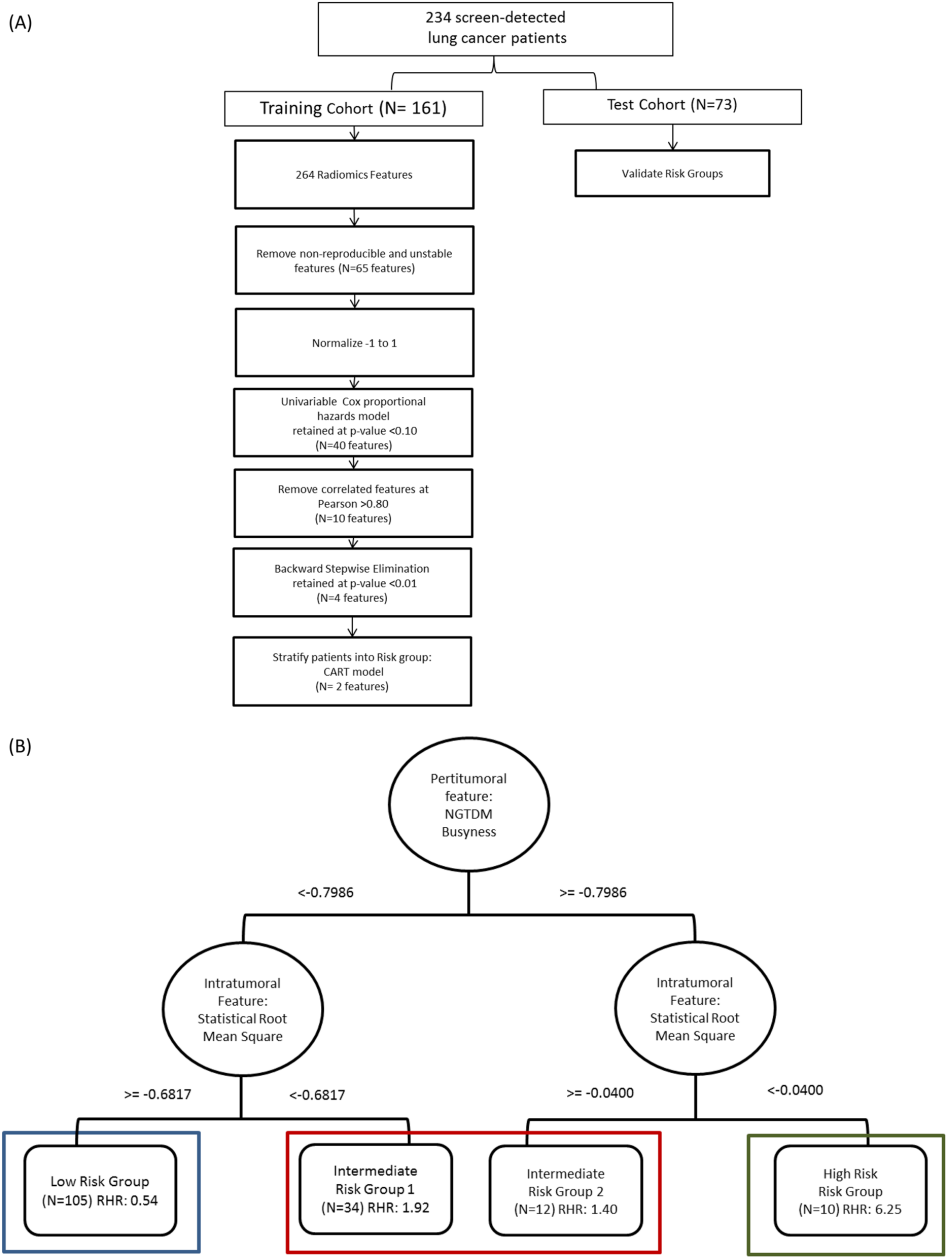
Identification of risk groups based on peritumoral and intratumoral features (**A**) Statistical analysis pipeline for radiomics feature selection. (**B**) The tree structure of the classification and regression tree analysis (CART) which identified four risk groups based on two radiomics features, in which we combined the two intermediate risk groups.

The four remaining radiomic features were subjected to Classification And Regression Tree (CART) analysis in the training cohort and based on 2 radiomic features (NGTDM Busyness and Statistical Root Mean Square). CART analysis classified patients into four risk groups: low-risk, intermediate-risk-1, intermediate-risk-2, and high-risk (Supplemental Fig. 1). The four risk groups were reduced to three risk groups by combining the two intermediate-risk groups (Fig. 1B) and 3 risk groups from the CART model was replicated in the test cohort.

**Figure 2:**
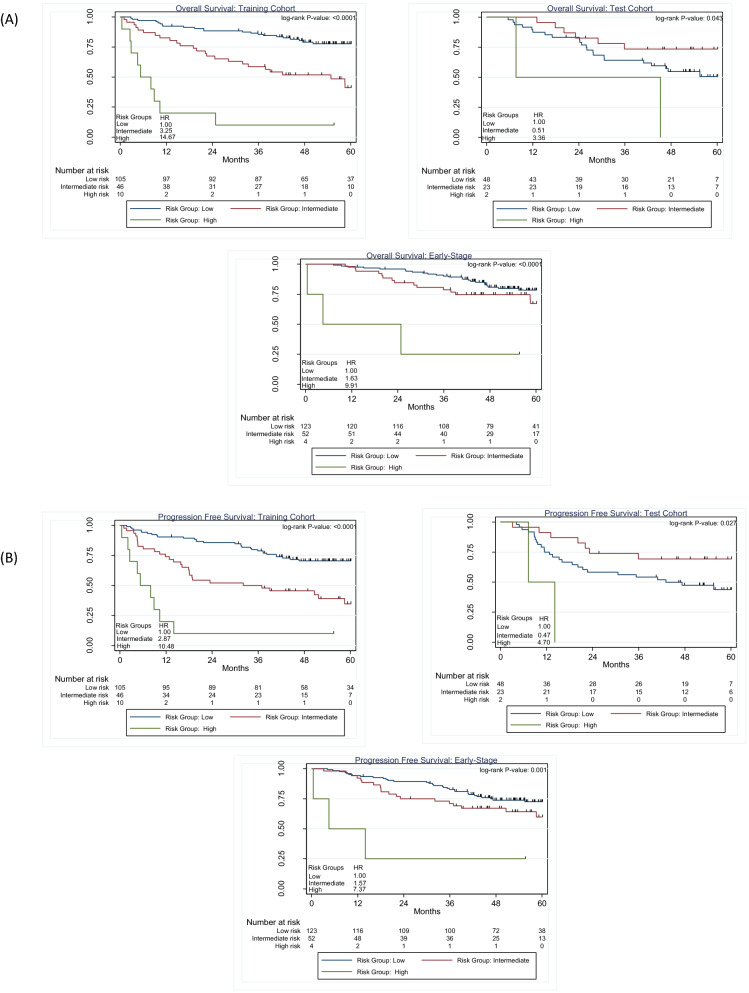
Risk-groups associated with overall survival for training and test cohorts and among early stage patients. Across the training and test cohort as well as in early-stage the high-risk group had a significantly worse outcome in OS (**A**) and PFS (**B**).

In training cohort, the high-risk group (Fig. 2A) was associated with extremely poor Overall Survival (OS) (Hazard Ratio (HR) = 14.67; 10% 2.5-year OS and 0% 5-year OS, log-rank *P* < 0.0001) versus the intermediate (HR = 3.25; 63% 2.5-year OS and 41% 5-year OS) and low-risk group (HR = 1.00; 89% 2.5-year OS and 78% 5-year OS). In the test cohort, the high-risk group was associated with extremely poor OS (HR = 3.35; 50% 2.5-year OS and 0% 5-year OS, log-rank *P* = 0.043) versus the low-risk group (HR = 1.00, 68% 2.5 year OS and 51% 5-year OS) (Fig. 2A). Similar findings were observed for Progression Free Survival (PFS) (Fig. 2B).

### Patient characteristics of the three risk groups in the total data set (N = 234)

There were no statistically significant differences between the three risk groups by age, smoking status, number of pack-years smoked, self-reported COPD, and family history of lung cancer, histological subtypes, and treatment (Table 2). However, there were statistically significant differences across the risk groups for sex (*P* = 0.04) and stage of disease (*P* = 0.001). Specifically, 92% of the patients in the high-risk group in were male vs. 54% in the low-risk group (*P* = 0.04) (Table 2). In term of lung cancer stage, 33% of the patients in the high-risk group had lung cancer early-stage vs. 80% in the low-risk group (*P* = 0.001).

**Table 2:** Patient characteristics of the three risk groups in the total data set (N = 234).

Characteristics	Low risk group (N = 153)	Intermediate risk group (N = 69)	High risk (N = 12)	*P* value^1,2,3^
Age, mean (sd)	63.1 (4.9)	65.2 (5.4)	63.7 (3.4)	0.07
Female	63.1 (4.9)	64.7 (5.6)	64	
Male	63.8 (4.9)	65.6 (5.4)	63.6 (3.6)	
Sex, N (%)				**0.04**
Female	71 (46%)	29 (42%)	1 (8%)	
Male	82 (54%)	40 (58%)	11 (92%)	
Smoking, N (%)				0.43
No	66 (43%)	31 (45%)	3 (25%)	
Yes	87 (57%)	38 (55%)	9 (75%)	
Pack-years, mean (sd)	63.2 (23.8)	66.5 (23.9)	74.0 (26.7)	0.95
FH of lung cancer				0.49
No	115 (75%)	47 (68%)	8 (67%)	
Yes	38 (25%)	22 (32%)	4 (33%)	
Self-reported history of COPD				0.19
No	140 (92%)	59 (86%)	12 (100%)	
Yes	13 (8%)	10 (14%)	0 (0%)	
Histology				0.27
Adenocarcinoma-BAC^4^	91 (59%)	36 (52%)	3 (25%)	
Squamous	26 (17%)	13 (19%)	5 (42%)	
Small cell	5 (3%)	4 (6%)	1 (8%)	
Other NOS	31 (20%)	16 (23%)	3 (25%)	
Treatment				0.13
Surgical	125 (82%)	50 (72%)	7 (58%)	
Chemotherapy/Other	13 (8%)	7 (10%)	1 (8%)	
Radiation therapy	15 (10%)	12 (17%)	4 (33%)	
Staging				**0.001**
Early stage (I and II)	123 (80%)	52 (75%)	4 (33%)	
Late stage (III and IV)	30 (20%)	17 (25%)	8 (67%)	
Baseline screening				0.06
Positive (T0+)	111 (73%)	41 (59%)	6 (50%)	
Negative (T0−)	42 (27%)	28 (41%)	6 (50%)	
2.5-year overall survival rate, %				** < 0.001**
Training	89%	63%	10%	
Test Cohort	68%	78%	50%	
5-year overall survival rate, %				** < 0.001**
Training	77%	41%	n/a	
Test Cohort	51%	73%	n/a	

Abbreviations: sd = standard deviation; FH = family history; Pack-years = packs smoked/day x years smoked; COPD = chronic obstructive pulmonary disease; NOS = not otherwise specified;

^1^ P-value obtained from Chi-squared for categorical variables.

^2^ P-value obtained from Anova for continuous variables.

^3^ P-value obtained from Log-rank for survival variables.

^4^ BAC and adenocarcinoma were combined into one group.

### Survival analyses among early stage patients

Among all early-stage patients, the high-risk group was associated with a significantly decreased OS (HR = 9.91; 25% 2.5-year and 0% 5-year OS, log-rank *P* < 0.0001) versus the low-risk group (HR = 1.00; 93% 2.5-year and 78% 5-year OS) (Fig. 2A). Similar results were found for PFS (Fig. 2B).

### Validation dataset

**Figure 3:**
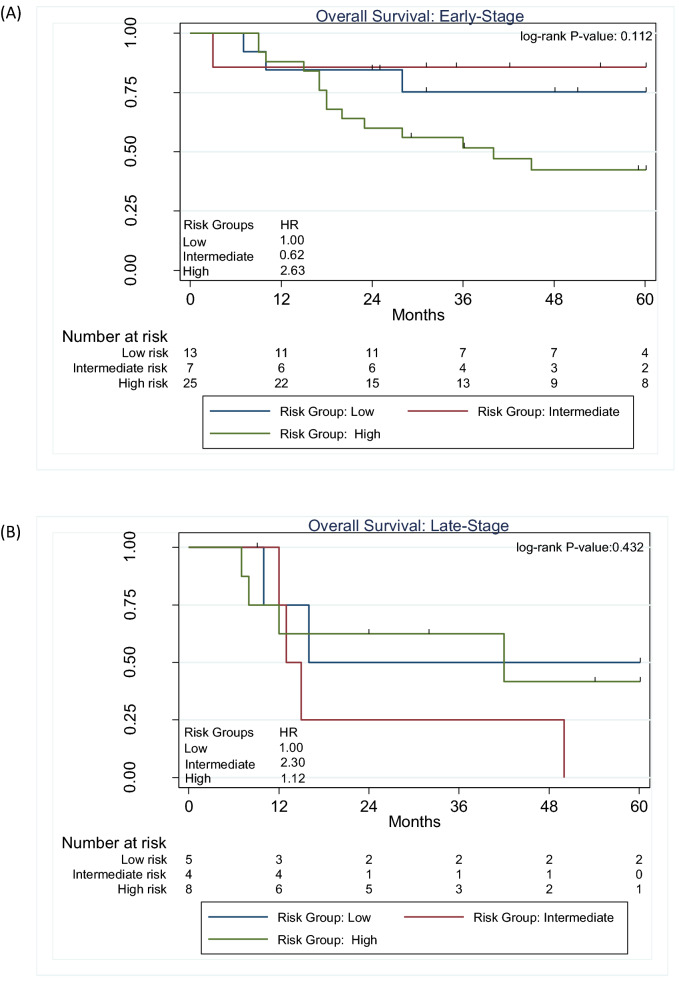
Overall survival for the risk patient risk groups among non-screen detected adenocarcinoma lung cancers (**A**) and for early-stage (**B**) for late-stage patients.

Using non-screen detected we attempted to replicate the risk groups obtained from the CART model. Among early-stage adenocarcinoma lung cancers (Fig. 3A), the high-risk group was associated with worse OS (HR = 2.63; 56% 2.5-year and 42% 5-year OS, log-rank *P* = 0.112) compared to the low-risk group (HR = 1.00; 75% 2.5-year and 75% 5-year OS). Among late-stage patients (Fig. 3B), the risk groups were not associated with survival (log-rank *P* = 0.432*).*

### Multivariable analyses

Multivariable Cox regression models were used to adjust for potential confounding factors including sex, treatment, and stage. In the training cohort, the high-risk group was associated with an elevated hazard ratio (OS: HR = 9.71; 95% Confidence Interval: [3.85, 24.48] and PFS: HR = 5.68; 95% Confidence Interval: [2.32, 13.93] ) when compared to intermediate and low-risk groups (Tables 3 and 4). In the test cohort, the high-risk group yielded an increased hazard ratio in PFS when compared to intermediate and low-risk groups (PFS: HR = 2.02; 95% Confidence Interval: [0.34, 11.99]). Among all patients, the high-risk group was associated with a significantly elevated hazard ratio (OS: HR = 5.16; 95% Confidence Interval: [2.34, 11.37] and PFS: HR = 4.02; 95% Confidence Interval: [1.89, 8.56]) when compared to the intermediate and low-risk group. For OS and PFS analysis, smoking status, sex, nor baseline screening were associated with our model.

**Table 3:** Multivariable Cox proportional hazards models for overall survival in the training and test cohorts.

Characteristic	**All Patients**^**2**^** HR (95% CI)**	**Training Cohort HR (95% CI)**	**Test Cohort HR (95% CI)**
Risk group			
Low	1.00 (Reference)	1.00 (Reference)	1.00 (Reference)
Intermediate	1.55 (0.95, 2.53)	**2.41 (1.28, 4.56)**	0.46 (0.15, 1.43)
High	**5.16 (2.34, 11.37)**	**9.71 (3.85, 24.48)**	0.93 (0.14, 6.33)
Sex			
Female	1.00 (Reference)	1.00 (Reference)	1.00 (Reference)
Male	1.46 (0.88, 2.41)	**2.41 (1.18, 4.94)**	1.13 (0.46, 2.74)
Smoking status			
Former	1.00 (Reference)	1.00 (Reference)	1.00 (Reference)
Current	1.13 (0.72, 1.80)	0.96 (0.53, 1.75)	1.71 (0.68, 4.34)
Baseline screening (T0)			
Positive (T0+)	1.00 (Reference)	1.00 (Reference)	1.00 (Reference)
Negative (T0−)	1.19 (0.72, 1.97)	0.84 (0.44, 1.61)	2.55 (0.94, 6.96)
Stage			
I and II	1.00 (Reference)	1.00 (Reference)	1.00 (Reference)
III and IV	**2.84 (1.58, 5.13)**	**4.42 (1.99, 9.82)**	1.68 (0.55, 5.11)
Histology			
Adenocarcinoma-BAC^1^	1.00 (Reference)	1.00 (Reference)	1.00 (Reference)
Squamous	1.31 (0.70, 2.46)	1.33 (0.85, 3.03)	1.19 (0.37, 3.84)
Small Cell	1.80 (0.79, 4.11)	2.21 (0.69, 7.08)	2.32 (0.62, 8.64)
Other NOS	1.18 (0.67, 2.06)	1.49 (0.71, 3.10)	2.16 (0.70, 6.73)
Treatment			
Surgical	1.00 (Reference)	1.00 (Reference)	1.00 (Reference)
Chemotherapy/Other	**4.36 (2.17, 8.75)**	**3.37 (1.24, 9.15)**	**5.31 (1.57, 17.91)**
Radiation	**3.29 (1.72, 6.29)**	1.55 (0.64, 3.76)	**13.52 (3.72, 49.12)**
Harrell's C index	0.79	0.83	0.81

Abbreviations: HR: Hazard Ratios; NOS: Not otherwise specified;

Data in parentheses are 95% CIs.

^1^BAC and adenocarcinoma were combined into one group.

^2^ “All patients” combines the training and test sets into a single cohort.

**Table 4:** Multivariable Cox proportional hazards models for progression free survival in the training and test cohorts.

Characteristic	All Patients HR (95% CI)	Training Cohort HR (95% Cl)	Test Cohort HR (95% CI)
Risk Group			
Low	1.00 (Reference)	1.00 (Reference)	1.00 (Reference)
Intermediate	1.42 (0.92, 2.22)	**2.04 (1.16, 3.59)**	**0.34 (0.12, 0.98)**
High	**4.02 (1.89, 8.56)**	**5.68 (2.32, 13.93)**	2.02 (0.34, 11.99)
Sex			
Female	1.00 (Reference)	1.00 (Reference)	1.00 (Reference)
Male	0.92 (0.60, 1.42)	1.29 (0.72, 2.31)	0.60 (0.27, 1.32)
Smoking Status			
Former	1.00 (Reference)	1.00 (Reference)	1.00 (Reference)
Current	1.21 (0.79, 1.85)	0.98 (0.57, 1.69)	1.77 (0.74, 4.23)
Baseline Screening			
Positive (T0+)	1.00 (Reference)	1.00 (Reference)	1.00 (Reference)
Negative (T0−)	1.47 (0.94, 2.31)	1.02 (0.57, 1.83)	**2.60 (1.15, 5.90)**
Stage			
I and II	1.00 (Reference)	1.00 (Reference)	1.00 (Reference)
III and IV	**3.54 (2.07, 6.04)**	**4.93 (2.45, 9.91)**	1.42 (0.55, 3.69)
Histology			
Adenocarcinoma-BAC^1^	1.00 (Reference)	1.00 (Reference)	1.00 (Reference)
Squamous	1.03 (0.60, 1.87)	1.06 (0.50, 2.26)	1.12 (0.36, 3.49)
Small Cell	1.49 (0.68, 3.25)	2.31 (0.75, 7.13)	1.49 (0.41, 5.37)
Other NOS	1.05 (0.63, 1.74)	0.95 (0.48, 1.89)	2.31 (0.86, 6.21)
Treatment			
Surgical	1.00 (Reference)	1.00 (Reference)	1.00 (Reference)
Chemotherapy/Other	**3.48 (1.86, 6.51)**	**3.13 (1.25, 7.87)**	**5.09 (1.75, 14.80)**
Radiation	**2.37 (1.29, 4.35)**	1.60 (0.69, 3.69)	**8.56 (2.51, 29.13)**
Harrel’s C statistics	0.79	0.81	0.80

### Performance metrics: Harrell’s c index and areas under the curve (AUROC)

**Figure 4:**
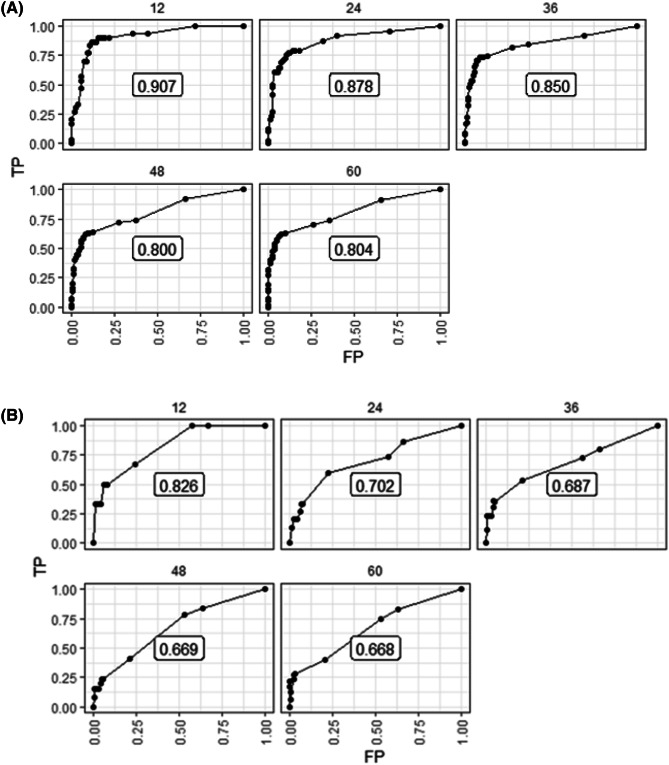
Time-dependent AUC plot of the multivariable model for overall survival for (**A**) all patients and (**B**) early-stage patients.

The discrimination performance of the multivariable model was estimated using the Harrell’s C index. The multivariable model showed a better discrimination capability with a higher C indices in the training and test cohort when analyzing OS (C-index: 0.83 and 0.81, training and test cohort respectively) compared to PFS (C-index: 0.81 and 0.80, training and test cohort respectively). Using the multivariable models, time-dependent areas under the curve (AUROC) for OS were generated and produced an AUROC of 0.878 at 2-years and 0.800 for 4-years. Among early-stage patients, the AUROC was 0.702 for 2-years and 0.669 and 4-years (Fig. 4). Similar results were found for PFS (Supplemental Fig. 3).

### Radiogenomics analysis

**Figure 5:**
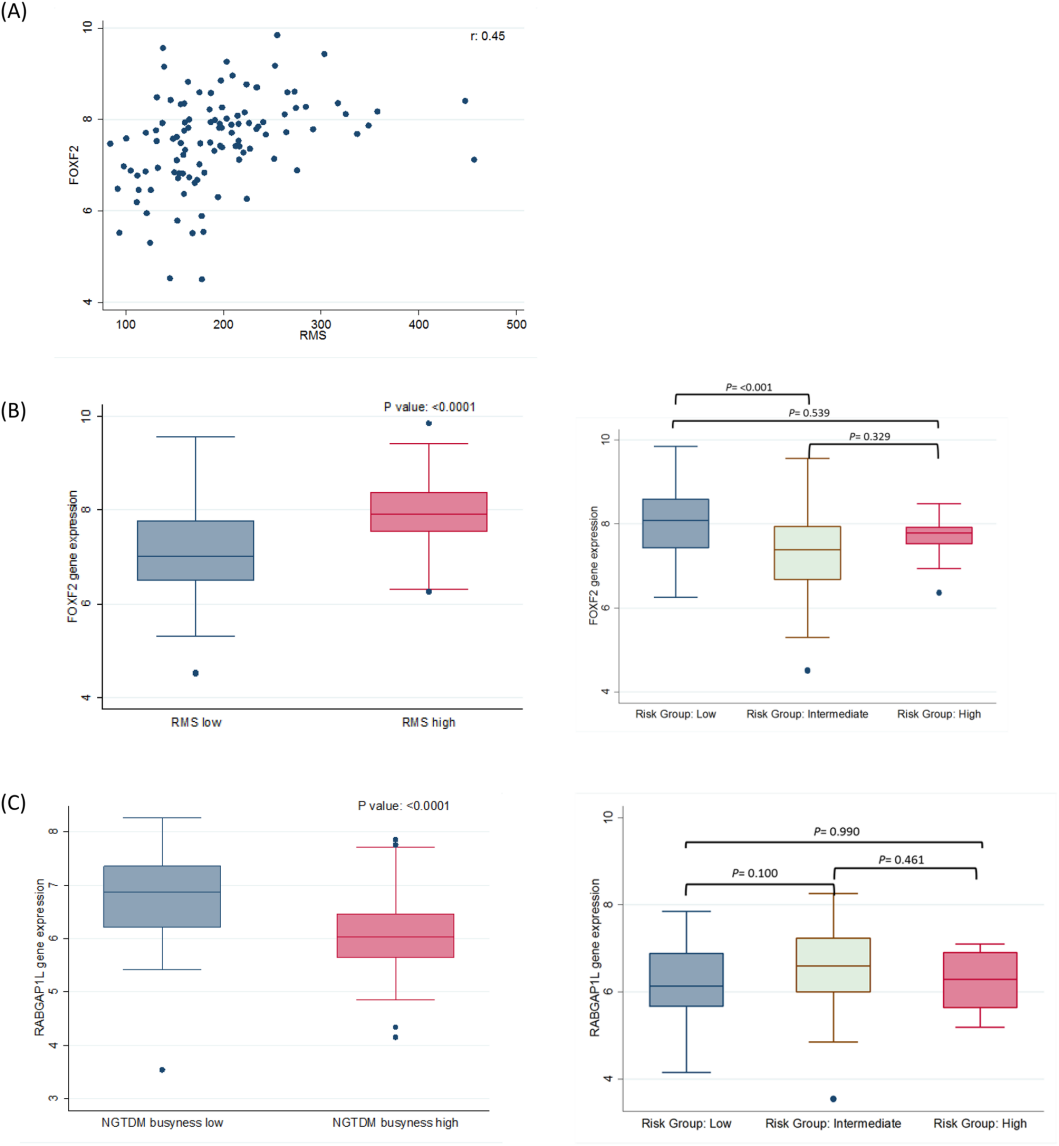
The association between radiomics gene expression. (**A**) Correlation between intratumoral RMS radiomic feature and *FOXF2*. (**B**) *FOXF2* expression by dichotomizing RMS at the median and FOXF2 expression by the three patient risk groups. (**C**) *RABGAP1L* expression by dichotomizing NGTDM busyness at the median and NGTDM busyness expression by the three patient risk groups.

From the radiogenomic analyses, FOXF2 and LOC285043 were found to be significantly associated with RMS feature in both the pair wise analysis and correlation analysis (Supplementary Table 3). Stage was not significantly associated with the two most informative radiomics features (*P* = 0.6828 for RMS and *P* = 0.7905 for NGTDM Busyness). From the correlation analyses, four genes were positively significantly correlated with the RMS radiomic feature *FOXF2*, *TBX4*, *LOC285043* and *TM4SF18.* Among these four genes, *FOXF2* had the highest correlation (r = 0.45) with RMS while the other genes were correlated at r = 0.44 (Fig. 5A). In the pair-wises analyses where RMS was dichotomized at the median value, six genes were significantly different the RMS high and RMS low. The mean value of *FOXF2* was significantly higher for RMS high vs. RMS low (mean = 7.1050 [SD = 1.0664] vs. 7.9798 [0.7054], p < 0.001) (Fig. 5B). When we generated the risk groups in the radiogenomics dataset, FOXF2 expression was significantly lower for the intermediate-risk group vs. low-risk group (mean = 7.2408 [SD = 1.0755] vs. 8.0287 [0.7524], p < 0.001). Among the three risk groups, the intermediate-risk had the lower FOXF2 expression when compared to the high-risk and low-risk group. Although FOXF2 expression was lower for the high-risk group vs. low-risk group (mean = 7.6819 [SD = 0.5879] vs. 8.0287[0.7521]), the difference was not statistically significant (p = 0.539).

No genes were significantly correlated with NGTDM busyness. In the pairwise analyses where NGTDM busyness was dichotomized at the median, three genes were significantly different between NGTDM busyness high vs. NGTDM busyness low: *RABGAP1L, LOC101928674*, and *LDLRAD4-AS1*. *RABGAP1L* expression was significantly higher NGTDM busyness low vs NGTDM busyness high (mean = 6.7612 [SD = 0.8313] vs. 6.1066 [0.8200], *P* < 0.001). For the risk groups, *RABGAP1L* expression was higher for the intermediate-risk group vs. low-risk group (mean = 6.5938 [SD = 0.8866] vs. 6.2120 [0.8981], *P* = 0.100). *RABGAP1* expression was higher for the high-risk group vs. low-risk group (mean = 6.2534 [SD = 0.6919] vs. 6.2120 [0.8981]), the difference was not statistically significant (*P* = 0.990). Stage was not significantly associated with the two most informative radiomics features (*P* = 0.6828 for RMS and *P* = 0.7905 for NGTDM Busyness) nor with the two most informative genes (*P* = 0.7767 for FOXF2 and *P* = 0.7928 for RABGAP1L).

## Discussion

Predictive biomarkers that identify aggressive cancers from those that are either indolent, or at lower-risk of poor survival outcomes, are a critical unmet need in the lung cancer screening setting. In this study, we utilized peritumoral and intratumoral radiomic features to generate a model that is able to detect vulnerable group of screen-detected early stage lung cancer patients that have high-risk of experiencing poor survival outcomes. Specifically, we identified a model that contained two radiomics features, one peritumoral and one intratumoral, which stratified patients into three risk-groups: low-risk, intermediate-risk, and high-risk. The model identified a vulnerable group early-stage patients with worse OS (HR = 9.91; 25% 2.5-year and 0% 5-year OS) versus the low-risk group (HR = 1.00; 93% 2.5-year and 78% 5-year OS). The final model was validated in the test cohort and further replicated in a cohort of non-screen detected adenocarcinoma patients. Because disease stage was significantly different across the risk groups, we stratified the model by stage and found compelling results among early stage patients, which typically have very good survival outcomes. Among early stage patients, the high-risk group was associated with a worse OS (HR = 2.63; 56% 2.5-year and 42% 5-year OS) compared to the low-risk group (HR = 1.00; 75% 2.5-year and 75% 5-year OS).

Radiomics is a non-invasive approach that utilizes standard-of-care imaging to generate quantitative image features that can be used for risk prediction, diagnostic discrimination, prognostication, and to predict treatment response^14,15,21–23^. Prior studies have shown that peritumoral features, extracted from the area surrounding the tumor parenchyma, and intratumoral features, extracted from the area within the tumor, have prognostic and predictive utility in cancers such as lung, breast, brain, gastric, and head and neck^1,24–29^. For example, Dong et al. (29) developed an individualized nomogram using radiomic features from primary tumor and from the peritoneum to identify occult peritoneal metastasis among patients with advanced gastric cancer^29^. Using peritumoral features from contrast-enhanced magnetic resonance imaging, Braman et al. found that CoL1AGe entropy was associated with pathological complete response among breast cancer patients who are Her2 negative^26^. In, Xu et al. identified a radiomic score using features from the peritumoral region of hepatocellular carcinoma tumors that predicts microvascular involvement^30^. Cumulative, the evidence of our study and others have demonstrate the utility of using peritumoral features alone or in combination with intratumoral features.

In this study, we identified a highly informative peritumoral feature (NGTDM busyness) and a highly informative intratumoral feature (statistical RMS). NGTDM, a texture feature, captures intensity values of a neighborhood of pixels to characterize the difference between a center voxel within the neighborhood^31,32^. NGTDM parameters are coarseness, contrast, and busyness. Coarseness describes the granularity of an image, contrast relates to the dynamic range of intensity, and busyness relate to the rate of intensity change within an image^32^. NGTDM busyness has a high predictive power in differentiating between glioblastoma and primary central nervous system lymphoma^33^. Studies found that NGTDM busyness extracted from positron emission tomography (PET) is useful for discriminating benign from malignant solid pulmonary nodules^34^. Intensity-based features are derived from image histograms which represent the intensity distribution of the image. Parmar et al. 2015 showed a correlation between intensity feature and patient survival in lung and head and neck cancer. Statistical RMS, an intensity feature, is a first-order statistic that calculates the root mean square of the voxel’s intensity value^31,35^. A previous study showed that statistical RMS was able to predict pathological response after chemoradiation in non-small cell lung cancer (NSCLC), by identifying gross residual response^35^. Statistical RMS combined with other intensity statistical features was able to distinguish bladder tumor tissue from other normal tissues in fluorodeoxyglucose-positron emission tomography (FDG-PET) scan^36^.

The radiogenomics analyses revealed that the most informative intratumoral radiomic feature, RMS, was significantly associated with expression of *FOXF2* and *LOC285043* which is an uncharacterized gene^37^. We observed a trend for lower expression of FOXF2 in intermediate and high-risk groups versus the low-risk group. However, this trend in high and low-risk group was not statistically significant. *FOXF2* is expressed in the lung and functions as an activator or inhibitor of gene transcription^38^ and up-regulation of *FOXF2* expression induces EMT, migration, invasion and metastasis in breast cancer^39^. To support our findings that low expression of FOXF2 is a negative prognostic factor, a prior study demonstrated that patients with stage I NSCLC who had low FOXF2 expression had significantly shorter overall survival compared to patients with high FOXF2 expression^40^. *RABGAP1L* was found to be significantly associated with NGTDM Busyness in the pairwise analyses and we revealed that the intermediate and high-risk groups had higher expression of *RABGAP1L* versus the low-risk group. At present, there is no known role for *RABGAP1L* in lung cancer. However, *RABGAP1L* has been shown to deregulate the tyrosine-kinase signaling pathway in acute myeloid leukemia^41^ and regulates the activity of GTPases which is essential to transport cell adhesion proteins and migrating cells^42^.

We acknowledge some limitations of this study. First, the sample size is somewhat modest because we utilized lung cancer cases with specific inclusion/exclusion criteria from the NLST. Then when we split the available number of cases into training and test cohorts based on a 70:30 ratio; the resulting sample sizes likely attributed to the poorly calibrated model based on its ability to predict 5-years survival outcomes in the training and test cohorts. However, we applied a rigorous feature reduction approach to eliminate correlated and non-reproducible features and utilized a backward reduction approach to identify a parsimonious model containing the most important features to reduce false positive findings. Although the overall population of lung cancer patients were treated heterogeneously; however, among the early stage patients, 92.74% of the patients had surgery as their only treatment. We also recognize that there were limited numbers of patients in the high-risk groups for these cohorts. Finally, our validation cohort was limited to patients with lung adenocarcinoma. Additional research is needed to validate the biological underpinnings of these features.

The results from our analyses produced a parsimonious radiomic model that identified a vulnerable subset of screen-detected lung cancers that are associated with poor outcome. These findings could support more aggressive treatment and follow-up for such high-risk patients. Nonetheless, additional research will be needed to inform the potential translational implications of these findings, to fully elucidate the biology these high-risk screen-detected tumors, to assess whether these findings are consistent across screening trials and cohorts, and how best to personalize cancer management in these vulnerable patients.

## Methods

### NLST study population

Deidentified data and LDCT images were accessed from the National Cancer Institute (NCI) Cancer Data Access System (CDAS)^43^. The NLST study design and main findings have been described previously^1,28^. NLST eligibility criteria included current and former smokers aged 55‐74 years with a minimum 30 pack‐years smoking history and former smokers had to have quit within the past 15 years.

Based on the schema described in Schabath et al.^44^, this analysis considered 321 NLST participants who had a negative or positive baseline screening (T0) result and were diagnosed with a screen-detected, incidental lung cancer on follow-up screening intervals 12 (T1) or 24 (T2) months after T0. Positive screens were defined as abnormalities on baseline screens or at follow-up screens that were new, stable or evolved. Negative screens were defined as a computerized tomography (CT) scan with no abnormalities, minor abnormalities, or significant abnormalities not suspicious for lung cancer. Among the 321 lung cancer patients, 196 had a baseline positive screen that was not diagnosed as lung cancer but evolved and diagnosed as lung cancer at T1 or T2 screening intervals. The remaining 125 lung cancer patients had a baseline negative screen result but developed a nodule that was diagnosed as lung cancer at either T1 or T2. Lung cancer patients who had multiple nodules at time of their diagnosis were excluded (N = 58) since we are unable to verify which nodule(s) were cancer. Complications with segmentation, such as a nodule attached to lung wall, led to 29 patients being excluded. The final dataset of 234 screen-detected lung cancers were randomly split into a training cohort (N = 161) and a test cohort (N = 73).

### Non-screen detected lung cancer validation dataset

The radiomics data were further validated for OS in a prior published dataset was comprised of 62 adenocarcinoma patients who underwent surgical resection as first course therapy at the Moffitt Cancer Center and had pre-surgery CTs within 2 months prior surgery^45^.

### Radiogenomics dataset

A previously described dataset^46^ of surgically resected adenocarcinoma lung cancers (N = 103) who had pre-surgery CTs and gene expression data was used to identify potential biological underpinnings of the final two informative radiomic features (RMS and NGTDM Busyness). The gene expression data were IRON-normalized and batch-corrected for RNA quality Pathway and Gene Ontology Enrichment using Clarivate Analytics MetaCore^46^.

### Radiomics

Nodule identification and tumor segmentation is described elsewhere^15^. The tumor mask images (i.e., tumor delineations) were imported into in-house radiomic feature extraction toolboxes created in MATLAB® 2015b (The Mathworks Inc., Natick, Massachusetts) and C+ + (https://isocpp.org). Using cubic interpolation, the images were resampled to a single voxel spacing of 1 mm × 1 mm × 1 mm to standardize spacing across all images. Hounsfield units (HU) in all images were resampled into fixed bin sizes of 25 HUs discretized from –1,000 to 1,000 HU.

Using standardized radiomic algorithms from the Image Biomarker Standardization Initiative (IBSI) v5^47^, a total of 264 radiomic features were extracted from semi-automatic segmented intratumoral region (n = 155) and from the peritumoral region (n = 109) 3 mm outside of tumor boundary. The peritumoral regions were generated as an extension of the tumor segmentations using morphological image processing operations as previously mentioned^48^. Peritumoral regions were bounded by a lung parenchyma mask to exclude the region of interest (ROI) outside of the lung parenchyma. Shape and size based peritumoral features were excluded as they explicitly describe (i.e., correlate) the intratumoral ROI. Only the most stable and reproducible intratumoral and peritumoral radiomic features that were previously found on another study of our group ^48^ were utilized for analyses to create repeatable models. Further details of the feature selection process are presented in the statistical analysis section below (Fig. 1A).

### Statistical analysis

Statistical analyses were performed using Stata/MP 14.2 (StataCorp LP, College Station TX), R Project for Statistical Computing (version 3.5.2), and R Studio (version 1.1.463). Fisher’s exact test was used to test the difference between the training and test cohorts for categorical variables and the Student’s *t*-test was used to test the difference between the training and test cohorts for continuous variables.

Overall survival (OS) and progression-free survival (PFS) were the main end-points for the analysis and were assessed from date of lung cancer diagnosis to the date of an event or last follow up. For OS, an event was defined as death and for PFS an event was established as death or progression of cancer. All survival data were right censored at 5-years. To generate a parsimonious radiomics model, we first performed univariable analyses using Cox Proportional Hazard regression and retained features with a *P* value < 0.10. Among the remaining features after univariable analyses, we removed features that were correlated based on Pearson’s correlation coefficient > 0.80. If two or more features were correlated based on an absolute Pearson’s correlation coefficient > 0.80, the feature with the smaller p-value from the univariable analyses was retained. The remaining radiomic features were subjected to backward elimination approach using a pre-specified more stringent *P* value < 0.01 for inclusion. Among the remaining covariates, Classification And Regression Tree analysis (CART) was used to stratify patients into risk groups. CART is a nonparametric data-mining tool that can identify hierarchical interactions and segment covariates into novel and meaningful terminal subgroups (i.e., nodes). The hazard ratios from the risk groups were generated using Cox Proportional Hazard regression. The risk groups were also analyzed by Kaplan Meier curves and log-rank tests. The risk groups based on the most informative radiomic features in the training cohort were validated in the test cohort and further replicated in the adenocarcinoma cohort. The Harrell’s concordance index (C-index) was used to evaluate the multivariable model. Time-dependent area under the receding operating curve (AUROC) analyses was used to assess accuracy of the Cox regression models at different time-points using R packages survival^49^, survminer^50^, and survivalROC^51^.

Using the radiogenomics dataset, analyses were conducted to determine if the two final radiomic features (RMS and NGTDM Busyness) were associated with the gene probesets using two different approaches: correlation and two-group analysis. For the correlation analysis, gene probesets were filtered and determined as statistically significant using the following criteria: Pearson’s correlation with a threshold |R|> 0.4, an expression filter with max expression of gene > 5, and an inter-quartile filter (IQR > log2 (1.2 FC)). For the two-group analyses, gene probesets were filtered and determined as significant using the following criteria based on a Student’s *t* test p < 0.001 and mean log fold-change between high and low prognostic radiomic feature oflfc > log2 (1.4 FC). The significant probesets from the two-group analyses were intersected yielding a final list of probesets significantly associated with the most informative radiomic feature. ANOVA and Tukey pairwise mean comparison was performed to analyzed gene expression across the risk groups.

## Supplementary information

Supplementary file1 (DOCX 129 kb)

## Data Availability

The datasets used and analyzed during the current study are available from the corresponding author on reasonable request.
