# Supplementary material for: Peritumoral and intratumoral radiomic features predict survival outcomes among patients diagnosed in lung cancer screening
Source: Sci Rep. 2020 Jun 29;10:10528. doi: 10.1038/s41598-020-67378-8 (PMC7324394; doi:10.1038/s41598-020-67378-8)
Supplement: Supplementary file 1 — Supplementary file1 (DOCX 129 kb) [file 41598_2020_67378_MOESM1_ESM.docx]

**Peritumoral and intratumoral radiomic features predict survival outcomes among patients diagnosed in lung cancer screening**

Jaileene Perez-Morales^1^, Ilke Tunali^2, 3^, Olya Stringfield^2^, Steven Eschrich^4^, Yoganand Balagurunathan^2^, Robert J. Gillies^2^, Matthew B. Schabath^1,5*^

^1^Department of Cancer Epidemiology, H. Lee Moffitt Cancer Center and Research Institute, Tampa, Florida, USA; ^2^Department of Cancer Physiology, H. Lee Moffitt Cancer Center and Research Institute, Tampa, Florida, USA; ^3^Institute of Biomedical Engineering, Bogazici University, Istanbul, Turkey; ^4^Department of Biostatistics and Bioinformatics, H. Lee Moffitt Cancer Center and Research Institute, Tampa, Florida, USA; ^5^Department of Thoracic Oncology, H. Lee Moffitt Cancer Center and Research Institute, Tampa, Florida, USA

*Corresponding Author: Matthew B. Schabath, Ph.D.

H. Lee Moffitt Cancer Center and Research Institute,

12902 Magnolia Drive MRC-CANCONT, Tampa, FL 33612

Fax: 1-813-745-4150

Tel: 1-813-745-4150

E-mail: [matthew.schabath@moffitt.org](mailto:matthew.schabath@moffitt.org)

**Figure Legends**

**Supplemental Figure 1:** Identification of risk groups based on peritumoral and intratumoral features (A) The tree structure of the classification and regression tree analysis (CART) which identified four risk groups based on two radiomics features. Kaplan Meier survival curves and overall log-rank test in the training cohort (B) and test cohort (C).

**Supplemental Figure 2:** Kaplan Meier survival curves at risk in female (A) and for male (B) for overall survival for screen-detected lung cancer.

**Supplemental Figure 3:** Time-dependent AUC plot of the multivariable model for Progression Free Survival.


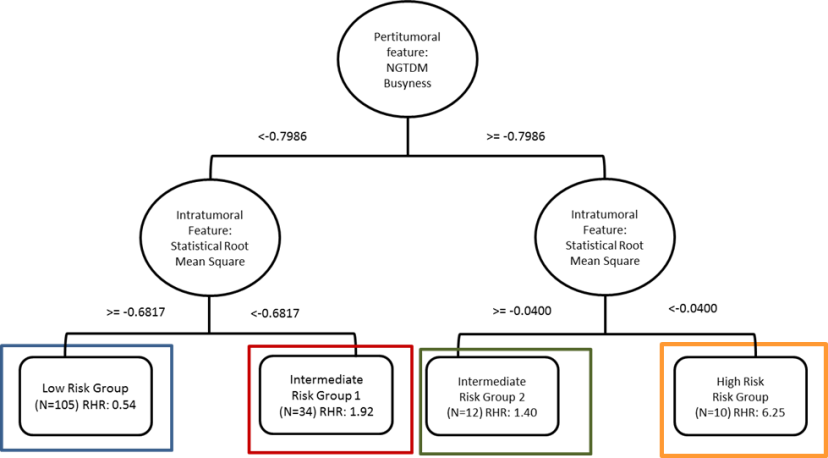
**Supplemental Figure 1.**

A.

B.

C.

**Supplemental Figure 2.**

A.

B.

**Supplemental Figure 3.**

All Patients


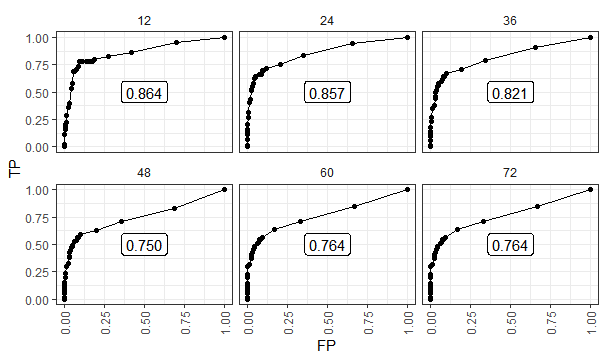


.

Early-Stage


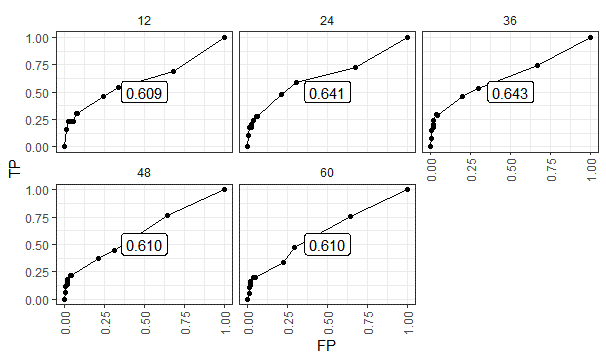


**Supplemental Table 1: Log-rank *P* values for the 40 stable features**

| Features | Hazard Ratio | *P* value | 95% Confidence Interval |
| --- | --- | --- | --- |
| Peritumoral Feature 19 | 0.27 | 2.64E-06 | [0.155, 0.465] |
| Peritumoral Feature 17 | 3.02 | 2.40E-05 | [1.809, 5.048] |
| Peritumroal Feature 102 | 3.86 | 3.79E-05 | [2.031, 7.343] |
| Intratumoral Feature 118 | 3.26 | 7.47E-05 | [1.816, 5.841] |
| Peritumoral Feature 126 | 2.77 | 9.17E-05 | [1.663, 4.621] |
| Peritumoral Feature 151 | 8.62 | 1.12E-04 | [2.890, 25.731] |
| Peritumoral Feature 129 | 0.37 | 1.18E-04 | [0.219, 0.610] |
| Intratumoral Feature 117 | 0.27 | 1.21E-04 | [0.137, 0.524] |
| Intratumoral Feature 131 | 3.05 | 1.32E-04 | [1.722, 5.411] |
| Intratumoral Feature 129 | 0.31 | 1.68E-04 | [0.171, 0.574] |
| Peritumoral Feature 101 | 4.25 | 2.04E-04 | [1.981, 9.136] |
| Intratumoral Feature 128 | 0.28 | 2.31E-04 | [0.140, 0.549] |
| Peritumoral Feature 35 | 2.80 | 3.30E-04 | [1.595, 4.900] |
| Intratumoral Feature 147 | 4.52 | 3.70E-04 | [1.971, 10.375] |
| Peritumoral Feature 92 | 3.70 | 3.71E-04 | [1.801, 7.616] |
| Peritumoral Feature 118 | 4.98 | 4.28E-04 | [2.038, 12.173] |
| Peritumoral Feature 112 | 19.43 | 5.42E-04 | [3.618, 104.341] |
| Intratumoral Feature 107 | 3.64 | 5.84E-04 | [1.744, 7.616] |
| Peritumoral Feature 148 | 0.39 | 6.10E-04 | [0.228, 0.668] |
| Peritumoral Feature 110 | 4.47 | 6.43E-04 | [1.892,10.572] |
| Intratumoral Feature 38 | 3.45 | 8.55E-04 | [1.666, 7.146] |
| Intratumoral Feature 105 | 3.96 | 9.54E-04 | [1.750, 8.948] |
| Peritumoral Feature 149 | 4.01 | 1.26E-03 | [1.724, 9.338] |
| Intratumoral Feature 109 | 4.25 | 1.48E-03 | [1.742, 10.385] |
| Intratumoral Feature 92 | 2.26 | 2.02E-03 | [1.347, 3.792] |
| Peritumoral Feature 38 | 3.84 | 2.43E-03 | [1.609, 9.165] |
| Peritumoral Feature 141 | 3.24 | 4.87E-03 | [1.429, 7.340] |
| Peritumoral Feature 36 | 0.30 | 5.50E-03 | [0.130, 0.703] |
| Peritumoral Feature 95 | 4.95 | 5.74E-03 | [1.591, 15.374] |
| Peritumoral Feature 131 | 2.31 | 6.77E-03 | [1.260, 4.228] |
| Peritumoral Feature 132 | 4.08 | 7.03E-03 | [1.468,11.326] |
| Peritumoral Feature 37 | 2.49 | 0.012 | [1.226, 5.072] |
| Peritumoral Feature 123 | 2.31 | 0.014 | [1.183, 4.524] |
| Peritumoral Feature 16 | 2.05 | 0.052 | [0.993, 4.227] |
| Peritumoral Feature 143 | 3.03 | 0.057 | [0.969, 9.484] |
| Intratumoral Feature 141 | 2.53 | 0.064 | [0.946, 6.765] |
| Intratumoral Feature 145 | 0.43 | 0.067 | [0.172, 1.061] |
| Peritumoral Feature 135 | 0.20 | 0.067 | [0.037, 1.120] |
| Peritumoral Feature 119 | 0.25 | 0.070 | [0.057, 1.122] |
| Intratumoral Feature 19 | 0.54 | 0.079 | [0.267, 1.074] |

**Supplemental Table 2: Hazard ratios for the stepwise backward elimination model**

| Features | Hazard Ratio | *P* value | 95% Confidence Interval |
| --- | --- | --- | --- |
| Peritumoral Feature 95 | 0.02 | 0.005 | [0.001, 0.298] |
| Peritumoral Feature 151 | 17.57 | 0.001 | [3.399, 90.857] |
| Intratumoral Feature 19 | 0.12 | 0.000 | [0.036, 0.383] |
| Peritumoral Feature 102 | 8.38 | 0.003 | [2.013, 34.911] |

| Supplementary Table 3: Summary of the statistically significant genes from the radio-genomic analyses | | | | | | |
| --- | --- | --- | --- | --- | --- | --- |
|  | **Correlation** | | | **Pairwise Comparison** | | |
| Intratumoral Feature: RMS | **Genes** | **r** | **Description** | **Genes** | ***P* value** | **Description** |
|  | *FOXF2** | 0.45 | Transcription factor, regulates EMT, and cell proliferation(50, 51) | *LOC285043** | 5.20E-07 | uncharacterized gene |
|  | *TBX4* | 0.44 | Required for cell proliferation(52) | *LOC105377335* | 4.01E-07 | uncharacterized gene |
|  | *LOC285043** | 0.44 | uncharacterized gene(44) | *FOXF2** | 2.55E-06 | Transcription factor, regulates EMT, and cell proliferation(50, 51) |
|  | *TM4SF18* | 0.44 | promotes invasion, motility, EMT, adhesion, and cell growth(53) | *PLEKHH2* | 1.91E-06 | cell adhesion and actin dynamics(54) |
| Peritumoral Feature: NGTDM busyness | **Genes** | **R** | **Description** | **Genes** | ***P* value** | **Description** |
|  | n/a | n/a | n/a | *RABGAP1L* | 7.00E-05 | promoted mTORC1 signaling when interacting with TUFT1(48) |
|  | n/a | n/a | n/a | *LOC101928674* | 1.24E-4 | uncharacterized gene |
|  | n/a | n/a | n/a | *LDLRAD4-AS1* | 1.30E-4 | LDLRAD4 antisense RNA 1 |
